# Building Integrated Ontological Knowledge Structures with Efficient Approximation Algorithms

**DOI:** 10.1155/2015/501528

**Published:** 2015-10-13

**Authors:** Yang Xiang, Sarath Chandra Janga

**Affiliations:** ^1^Department of Biomedical Informatics, The Ohio State University, Columbus, OH 43210, USA; ^2^Department of BioHealth Informatics, Indiana University-Purdue University Indianapolis, Indianapolis, IN 46202, USA

## Abstract

The integration of ontologies builds knowledge structures which brings new understanding on existing terminologies and their associations. With the steady increase in the number of ontologies, automatic integration of ontologies is preferable over manual solutions in many applications. However, available works on ontology integration are largely heuristic without guarantees on the quality of the integration results. In this work, we focus on the integration of ontologies with hierarchical structures. We identified optimal structures in this problem and proposed optimal and efficient approximation algorithms for integrating a pair of ontologies. Furthermore, we extend the basic problem to address the integration of a large number of ontologies, and correspondingly we proposed an efficient approximation algorithm for integrating multiple ontologies. The empirical study on both real ontologies and synthetic data demonstrates the effectiveness of our proposed approaches. In addition, the results of integration between gene ontology and National Drug File Reference Terminology suggest that our method provides a novel way to perform association studies between biomedical terms.

## 1. Introduction

In recent years, ontologies are becoming increasingly important in knowledge engineering. Generally speaking, an ontology is a collection of concepts and their relations. It has wide applications in computer science and life science. For example, in computer science, semantic web uses web ontology language (OWL) to represent knowledge bases [1]. In life sciences, numerous important data structures and tools are built on ontologies. One of the most popular ontologies is gene ontology. Researchers use it frequently to measure the enrichment of gene clusters and to identify potential biomarkers. Two most famous ontology databases in the biomedical field are Unified Medical Language System (UMLS) [2] and NCBO BioPortal (https://bioportal.bioontology.org/). The former has more than 100 ontology datasets and the latter has more than 300 ontology datasets.

Although ontologies can be modeled as a directed graph, many ontologies are in fact hierarchical trees or have hierarchical tree-like structures. In the BioPortal website, users can find basic hierarchical properties of an ontology, such as the maximum depth and the maximum number of children. In the UMLS, the hierarchical structure of an ontology is documented in the “MRHIER.RRF” file with each line being a path from a term to its root. We can build a hierarchical tree from these paths by merging the common nodes starting from the root. Because the hierarchical structures of some ontologies are in fact directed acyclic graphs, the hierarchical tree may contain some duplicated concepts. To simplify our study, we treat them as independent concepts in this work.

An important knowledge discovery task is to identify knowledge associations. In life science, this task includes finding the associations between diseases and genes [3, 4] and between phenotypes and genotypes [5]. With the presence of ontologies, such a task has been extended from identifying the associations between terms to the associations between ontologies as a whole. For the latter, we should not only consider the term associations, but also the term associations in the context of their ontological structures. For example, if the parent and children of term *a* (*a* from ontology *A*) are similar to those of term *b* (*b* from ontology *B*), Then *a* may be a good choice to be associated with *b*.

Early studies on ontology integration relied on domain experts to manually set up the integration rules [6]. However, this approach cannot meet the ever increasing volume of ontology datasets. Automatic ontology integration methods have been developed to address this issue. However, as we will see in the discussions of Section 1.3, these methods are often heuristic or have not demonstrated the effectiveness in integrating a large volume of ontology datasets. Thus, our goal is to develop an ontology integration method that is able to deliver optimal or close-to-optimal solutions for integrating a large volume of ontology datasets (particularly from the biomedical domain). As discussed above, we focus on ontologies with hierarchical tree-like structures which are often available in the biomedical domain. In addition, we assume the ontology term closeness measurement is available. This assumption is reasonable because many applications are able to identify ontology term similarities via additional data sources. For example, a closeness matrix between two sets of biomedical terms can be generated by using UMLS knowledge discovery methods such as kDLS [7]. Our problem can be formally described as follows.

### 1.1. Problem Formulation

The basic ontology integration problem in our work can be formulated as follows. Given ontology tree structures *T*_*A*_ and *T*_*B*_ and a closeness matrix *M*_*AB*_, how can we efficiently generate an integrated ontology tree structure *T*_*AB*_ meeting the following two basic criteria?

(1) For any two vertices *x* and *y* in *T*_*A*_ (or *T*_*B*_), the lowest common ancestor LCA_*T*_*A*__(*x*, *y*) (or LCA_*T*_*B*__(*x*, *y*)) is contained by LCA_*T*_*AB*__(*x*, *y*).
(2) It holds that argmax_*T*_*AB*__ *f*(*T*_*AB*_) = ∑_*X*∈*V*(*T*_*AB*_)_ *M*_*AB*_(*X*). Here *M*_*AB*_(*X*) is the entry value in the closeness matrix for the corresponding two vertices (one from *T*_*A*_ and the other from *T*_*B*_) contained in the node *X*. *M*_*AB*_(*X*) = 0 if *X*, a node of *T*_*AB*_, contains only one vertex from *T*_*A*_ or *T*_*B*_.

**Figure 1:**
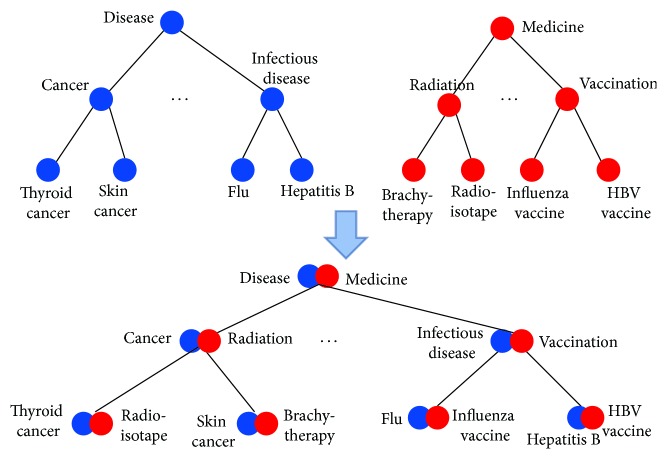
A simple example of integrating two hypothetical ontologies.

We name *f*_*T*_*AB*__ the cohesion function of the integrated ontology *T*_*AB*_ and its value is the overall cohesion score of integrating *T*_*A*_ and *T*_*B*_ into *T*_*AB*_. Correspondingly, we define function *g*(*T*_*A*_, *T*_*B*_) = max_*T*_*AB*__(∑_*v*∈*V*(*T*_*AB*_)_ *M*_*AB*_(*v*)) as the maximum cohesion function for integrating the ontologies *T*_*A*_ and *T*_*B*_ and its value is the maximum overall cohesion score (or, simply, maximum cohesion score). In a hierarchical ontology, the common part of any two terms can be described by their lowest common ancestor. For example, in Figure 1, flu and hepatitis B are both infectious diseases, and flu and cancer are both diseases. Thus, we use Criterion (1) to ensure that the basic logic of an ontology is preserved after integration.

**Figure 2:**
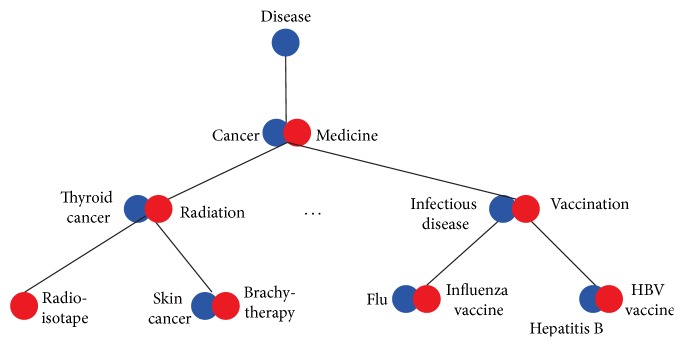
Another example of integrating the two ontologies in Figure 1. This integration violates Criterion (1).

An example of integrating two hypothetical ontologies that satisfy Criterion (1) is given in Figure 1. To facilitate the understanding of our problem definition, we also provide another example of integrating two ontologies in Figure 2. As we can see in Figure 2, the lowest common ancestor of nodes containing thyroid cancer and infectious disease is the node containing cancer instead of disease. We conclude that the integration is a violation of Criterion (1). In fact, we can easily see that there are multiple pairs of nodes with incorrect lowest common ancestors in Figure 2.

In Section 2.2, we will extend the basic problem definition to handle the integration of multiple (>2) ontologies. The two basic criteria will be extended correspondingly.

### 1.2. Main Contributions

We made the following major contributions in this work.

- We proposed a novel ontology integration problem that optimizes the cohesion function. We identified optimal structures in this problem and developed optimal as well as efficient approximation solutions for this problem.
- We extended the basic problem to handle the integration of large number of ontologies, and we developed both greedy and fast approximation algorithms for the extended problem.
- We studied the proposed algorithms on both real and synthetic datasets and confirmed their effectiveness in integrating large volume of ontology datasets.

### 1.3. Related Work

Automatic ontology generation and integration are desirable in many applications and have been studied in the past decade. Although available methods for automatic ontology generation produce ontologies from a given type of data, such as gene networks [8], textual data [9], dictionary [10], and schemata [11, 12], they do not contribute to the integration of different types of ontologies which will bring innovative results on annotation/knowledge reuse and association studies. To address this issue, a number of studies have been focused on ontology integration [13, 14] and its medical domain applications [15]. The ontology integration methods available and used in these works can be generally classified into three categories.

*Manually or Semiautomatic Setups*. In [6], the authors presented a methodology for ontology integration by custom-tailored integration operations which include algebraic-specified 39 basic operations and 12 nonbasic operations derived from them. The authors identified a set of criteria, such as modularize, specialize, and diversify each hierarchy, for guiding the knowledge integration. In [16], the authors designed a semiautomatic approach for ontology merging. The ontology developers will be assisted by the system and guided to tasks needing their interventions.

*Using Machine Learning Methods*. Reference [17] describes an ontology mapping system GLUE that uses machine learning techniques for building ontology mappings. Specifically, GLUE uses multiple learning strategies. Each type of information from the ontologies is handled by a different learning strategy. The authors demonstrate that GLUE works effectively on taxonomy ontologies. Similarly, [18] also used multistrategy learning in matching pair identification. However, the ontologies used in the experiments of [18] contain less than 10 nodes. Although [17] studied the integration of larger ontologies in the experiments, those ontologies contain only around 34 to 176 nodes, much smaller than many ontologies used in the biomedical field.

*Using Heuristic Approaches*. Many automatic ontology integration methods [19, 20] fall into this category. They perform ontology integrations by using heuristic approaches from different perspectives. For example, [20] uses heuristic policies for selecting axioms and candidate merging pairs. From a quite different angle, [19] uses view-based query for guiding the ontology integrations.

These methods have a few major weaknesses including (1) lack of a systematic measurement to quantify the goodness for the ontology integration; (2) being generally heuristic with no theoretical results to show that the proposed integration approach is globally optimal or close to optimal; (3) being not for integrating large volume of ontologies. These weaknesses motivated us to develop efficient and near optimal solutions for integrating large ontology datasets.

## 2. Methods

### 2.1. Integrating a Pair of Ontologies

In this section, we focus on the basic problem of integrating two ontologies as formulated in Section 1.1. We will prove optimal structures in the problem and propose an optimal and an efficient approximation solution for this problem. These solutions are also the basis for solving the problem of integrating a large number of ontologies as described in Section 2.2.

#### 2.1.1. Brutal-Force and Heuristic Solutions

Given Criteria (1) and (2), a brutal-force approach will pick up a best solution from all the solutions that start with integration involving at least one of the roots of the two ontology trees and iteratively integrate their descendants. Considering an extreme case where each ontology tree is a path of *n* vertices, we conclude that such a brutal-force approach needs to pick up a best solution from an exponential number of solutions. The brutal-force approach is clearly not acceptable for integrating large ontologies and may not even work for ontologies with only a few dozens of vertices.

A heuristic solution can be developed by following an idea similar to the above brutal-force approach. However, instead of trying all possibilities, the heuristic solution will greedily merge vertices following the topological order. When selecting a matching vertex for vertex *a* from ontology *T*_*A*_, the heuristic approach will greedily select a vertex *b* from allowable candidates in *T*_*B*_ and iteratively apply such selections to descendants of *a*. According to Criterion (1), if *a* is associated with *b*, then none of *a*'s descendants are allowed to be associated with vertices other than *b*'s descendants. In addition, if *a*'s child *a*′ is associated with *b*'s child *b*′ or its descendants, then none of *a*'s other children are allowed to be associated with *b*′ or its descendants any more. Given this, a greedy choice may very easily end up in a local optimum by choosing a best matching vertex at one step, while denying integrating opportunities of other vertices that may lead to a better final solution.

It is easy to see that the deeper a vertex being chosen for integration is, the more integration opportunities are lost. To alleviate such a situation, we propose a greedy approach by considering the relative depth (rdepth) of a chosen vertex with regard to an allowable vertex closest to the root. That is, given a vertex *a* from *T*_*A*_, a vertex *b* from *T*_*B*_ is chosen when *M*_*AB*_(*a*, *b*)/*β* ^rdepth(*b*)^ is maximized. When *β* = 1, the depth information does not take effective and when *β* = *∞*, each vertex will only be associated with an allowable vertex closest to the root.

Algorithm 1 describes the pseudocode of the heuristic integration. It starts by integrating virtual roots of the two ontologies. After that, the integration will be carried out iteratively from top to bottom by following Criterion (1) and the heuristic strategy described above. In the empirical study, we will see that the heuristic algorithm works better when the depth information is considered. However, in terms of the overall cohesion score, it is no match for our optimal and approximation solutions as described below.

#### 2.1.2. Optimal and Approximation Solutions

By dividing the integration of two trees into node merging and subtree integrations, we have identified optimal structures in the basic problem, as stated by Lemmas 1 and 2. These optimal structures make it possible for us to develop efficient algorithms (Algorithms 2 and 3) that achieve optimal or approximation solutions. In the following, we first describe the two important lemmas suggesting the optimal structures and their proofs before describing our proposed algorithms.

**Lemma 1 .**

Let *r*_*a*_ be the root of tree *T*_*A*_ and *r*_*b*_ the root of tree *T*_*B*_. Let *T*_*A*−*r*_*a*__ and *T*_*B*−*r*_*b*__ represent two sets of sub trees rooted at *r*_*a*_ of *T*_*A*_ and *r*_*b*_ of *T*_*B*_, respectively. *T*_*A*−*r*_*a*__ does not include *r*_*a*_ and *T*_*B*−*r*_*b*__ does not include *r*_*b*_. One has *g*(*T*_*A*_, *T*_*B*_) = max(*M*_*AB*_(*r*_*a*_, *r*_*b*_) + *g*(*T*_*A*−*r*_*a*__, *T*_*B*−*r*_*b*__), *g*(*T*_*A*_, *T*_*B*−*r*_*b*__), *g*(*T*_*A*−*r*_*a*__, *T*_*B*_)).

**Proof**

We can divide the integration of tree *T*_*A*_ with tree *T*_*B*_ into two cases according to the merging of their roots.

(1) The roots of *T*_*A*_ and *T*_*B*_ are merged together.
(2) The roots of *T*_*A*_ and *T*_*B*_ are not merged together.

For case (1), it is clear that the cohesion score is *M*_*AB*_(*r*_*a*_, *r*_*b*_) + *g*(*T*_*A*−*r*_*a*__, *T*_*B*−*r*_*b*__).

For case (2), we conclude that either *T*_*A*_ is integrated with *T*_*B*−*r*_*b*__ (*r*_*b*_ is out of integration) or *T*_*B*_ is integrated with *T*_*A*−*r*_*a*__ (*r*_*a*_ is out of integration). Otherwise, we will have a merged tree *T*_*AB*_ with two roots *r*_*a*_ and *r*_*b*_, a contradiction to the fact that *T*_*AB*_ is a tree. Therefore, the cohesion score is either *g*(*T*_*A*_, *T*_*B*−*r*_*b*__) or *g*(*T*_*A*−*r*_*a*__, *T*_*B*_).

Combining cases (1) and (2) and according to Criterion (2), we have

(1) gTA,TB=maxMABra,rb+gTA−ra,TB−rb,   kkgTA,TB−rb,gTA−ra,TB.

**Lemma 2 .**

Let *T*_*A*−*r*_*a*__ and *T*_*B*−*r*_*b*__ represent two sets of trees obtained by removing the root vertices *r*_*a*_ from *T*_*A*−*r*_*a*__ and *r*_*b*_ from *T*_*B*−*r*_*b*__. One has

(2) $$\begin{array}{c} g\left(T_{A-r_{a}},T_{B-r_{b}}\right)=max\left(\underset{\left(T_{x},T_{y}\right)\in R}{\sum }\left(g\left(T_{x},T_{y}\right)\right)\right). \end{array}$$

Here *T*_*x*_ ∈ *T*_*A*−*r*_*a*__, *T*_*y*_ ∈ *T*_*B*−*r*_*b*__, and *R* is a matching of trees in *T*_*A*−*r*_*a*__ with trees in *T*_*B*−*r*_*b*__.

**Proof**

**Figure 3:**
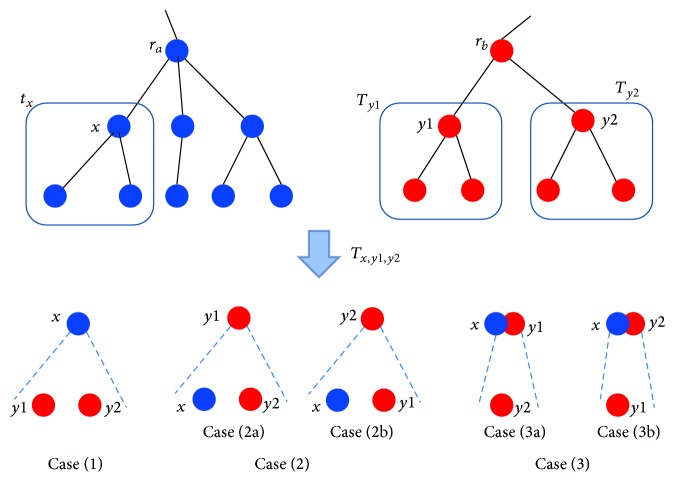
An illustration of three cases in the proof of Lemma 2.

To prove this lemma, we first prove that for any tree *T*_*x*_ ∈ *T*_*A*−*r*_*a*__, it can be integrated with no more than one tree in *T*_*B*−*r*_*b*__. We will prove this claim by contradiction. Assume there are two trees *T*_*y*_1__ ∈ *T*_*B*−*r*_*b*__ and *T*_*y*_2__ ∈ *T*_*B*−*r*_*b*__ and they integrate with a tree *T*_*x*_ ∈ *T*_*A*−*r*_*a*__ into an integrated tree *T*_*x*,*y*_1_,*y*_2__. There are three cases for the root *r* of *T*_*x*,*y*_1_,*y*_2__, as illustrated in Figure 3:

(1) *r* contains only the root of *T*_*x*_;
(2) *r* contains only the root of *T*_*y*_1__ or *T*_*y*_2__;
(3) *r* contains the root of *T*_*x*_ and the root of *T*_*y*_1__ or *T*_*y*_2__.

For case (1), the lowest common ancestor of the roots of *T*_*y*_1__ and *T*_*y*_2__ in the integrated tree *T*_*x*,*y*_1_,*y*_2__ will no longer contain their lowest common ancestor in *T*_*B*_, a contradiction to Criterion (1). For cases (2) and (3), the root of *T*_*y*_2__ (or the root of *T*_*y*_1__) will be the descendant of the root of *T*_*y*_1__ (or the root of *T*_*y*_2__) in the integrated tree *T*_*x*,*y*_1_,*y*_2__, a contradiction to Criterion (1). For integration involving more than two trees from *T*_*B*−*r*_*b*__, we can still follow the above procedure to reach contradictions. Thus, the claim is proven.

Without loss of generality, we can see that, for any tree *T*_*y*_ ∈ *T*_*B*−*r*_*b*__, it can be integrated with no more than one tree in *T*_*A*−*r*_*a*__. Therefore, the integration between *T*_*A*−*r*_*a*__ and *T*_*B*−*r*_*b*__ corresponds to a matching in a weighted bipartite graph in which two sets of nodes represent trees from *T*_*A*−*r*_*a*__ and *T*_*B*−*r*_*b*__, respectively, and edges represent corresponding cohesion scores. According to Criterion (2), *g*(*T*_*A*−*r*_*a*__, *T*_*B*−*r*_*b*__) = max(∑_(*T*_*x*_,*T*_*y*_)∈*R*_(*g*(*T*_*x*_, *T*_*y*_))) and we conclude that *g*(*T*_*A*−*r*_*a*__, *T*_*B*−*r*_*b*__) corresponds to the weight of a maximum weighted matching in the above bipartite graph.

Given Lemma 2, we can see that the following corollary is correct.

**Corollary 3 .**

Define *MaxMatch*(*X*, *Y*) = ∑_(*T*_*x*_,*T*_*y*_)∈*R*_ *g*(*T*_*x*_, *T*_*y*_), where *R* is a maximum weighted matching of trees in forests *X* and *Y* given *g*(*T*_*x*_, *T*_*y*_) for any tree pair (*T*_*x*_, *T*_*y*_) ∈ *X* × *Y*. One concludes that, for any two forests *T*_*A*_ and *T*_*B*_, *g*(*T*_*A*_, *T*_*B*_) = *MaxMatch*(*T*_*A*_, *T*_*B*_).

With Lemma 1 and Corollary 3, we are able to design an efficient dynamic programming algorithm achieving the global optimum for the ontology integration problem. The pseudocode for calculating the maximum cohesion score is described in Algorithm 2, which visits ontology vertices in reverse topological order when filling up the cohesion matrix.

At the end of Algorithm 2, the cohesion matrix is filled up with optimal cohesion scores and the maximum cohesion score is saved at entry (0,0), as described by Theorem 4.

**Theorem 4 .**

It holds that *cohesion*_*matrix*(*i*, *j*) = *g*(*T*_*A*−*i*_, *T*_*B*−*j*_) and *cohesion*_*matrix*(0,0) = *g*(*T*_*A*_, *T*_*B*_).

**Proof**

We will prove this theorem by mathematical induction.

Let |*V*(*T*_*A*_)| = *n* and |*V*(*T*_*B*_)| = *m*; it is easy to see that cohesion_matrix(*n*, *m*) is optimal because *n* and *m* correspond to leaf nodes in the topological order. Thus *T*_*A*−*n*_ and *T*_*B*−*m*_ are empty sets and cohesion_matrix(*n*, *m*) = *M*_*AB*_(*n*, *m*) = *g*(*T*_*n*_, *T*_*m*_).

When *i* = *n* and *j* < *m*, according to Lemma 1, to integrate *T*_*n*_ with *T*_*j*_, either *T*_*n*_ (the leaf vertex) is merged with *T*_*j*_ or *T*_*n*_ is integrated with *T*_*B*−*j*_. The maximum score of integrating *T*_*n*_ with *T*_*B*−*j*_ is available at the time cohesion_matrix(*n*, *j*) is being calculated because of the reverse topological visit. Thus, according to both Lemma 1 and Corollary 3, we conclude that *g*(*T*_*n*_, *T*_*j*_) = max(*M*_*AB*_(*n*, *j*), MaxMatch(*T*_*n*_, *T*_*B*−*j*_)) = cohesion_matrix(*n*, *j*). Similarly, we can conclude that *g*(*T*_*i*_, *T*_*m*_) = max(*M*_*AB*_(*i*, *m*), MaxMatch(*T*_*A*−*i*_, *T*_*m*_)) = cohesion_matrix(*i*, *m*).

When *i* < *n* and *j* < *m*, again, according to Lemma 1 and Corollary 3, we have *g*(*T*_*i*_, *T*_*j*_) = max(score, score_*a*_, score_*b*_) = cohesion_matrix(*i*, *j*). Due to the reserve topological visit, MaxMatch(*T*_*A*−*i*_, *T*_*B*−*j*_), MaxMatch(*T*_*A*−*i*_, *T*_*j*_), and MaxMatch(*T*_*i*_, *T*_*B*−*j*_) are available at the time cohesion_matrix(*i*, *j*) is being calculated.

Given the definition of *g*(*T*_*A*_, *T*_*B*_), Theorem 4 in fact proves that the proposed approach achieves the global optimum. However, the global optimal solution is built upon the maximum weighted matching (recall Corollary 3). As discussed in Section 2.1.3, the maximum weighted matching is time-consuming and therefore we propose an approximation solution in that section.

Although Algorithm 2 builds the cohesion matrix with optimal cohesion scores, it does not construct the integrated ontological knowledge structure. We may save the ontology integration details along with the cohesion scores. However, that will cost *O*(*n* ^3^) memory space (assuming each ontology has *O*(*n*) vertices) and significantly reduce the capacity of the algorithm in handling large ontology integrations. Quite interestingly, we find that it is not necessary to save the integration details in order to construct the integrated ontology. The construction can be done by a process reverse to the construction of cohesion matrix, as described in Algorithm 3.

Algorithm 3 uses the cohesion matrix constructed by Algorithm 2 and builds the integrated ontology tree still by following Lemma 1 and Corollary 3, but in a reverse way of Algorithm 2. The construction is performed in a Breadth-First fashion which uses a queue *q* to maintain triples. Each triple (*a*, *b*, *c*) is an association of three elements: *a* is the matched vertex from ontology *T*_*A*_; *b* is the matched vertex from ontology *T*_*B*_; and *c* is their parent on the merged ontology *T*_*AB*_. By following the basic idea of the proof of Theorem 4 we can show that Algorithm 3 builds an optimal integrated ontology with the cohesion matrix provided from Algorithm 2. We omit the proof for succinctness.

#### 2.1.3. Time Complexity Analysis and an Approximation Solution

Assume an ontology size is *O*(*n*). The cohesion matrix has *O*(*n* ^2^) entries to fill up. The computation for each entry is a matching whose time complexity depends on the implementation. The maximum weighted matching takes *O*(*n* ^3^) using the famous Hungarian algorithm [21], and although it achieves optimum, it is too costly for large ontologies. The maximal weighted matching, however, takes *O*(*n* ^2^log*n*) time and, more importantly, results in an overall *O*(*n* ^2^log*n*) time complexity for Algorithm 2. The analysis is given in the following. For each matching, the algorithm will access previously filled entries and each entry will be accessed only once and be involved only once in a sorting of *O*(log*n*) time. This is because each entry corresponds to two vertices whose cohesion score will be accessed when calculating the cohesion score of their parents. Thus, we conclude that the total time complexity of calculating the cohesion matrix using maximal weighted matching is *O*(*n* ^2^ + *n* ^2^log*n*) = *O*(*n* ^2^log*n*). Since building the new ontology has the same time complexity as building the cohesion matrix, this is also the total time complexity for integrating two ontologies by maximal weighted matching. The maximal weighted matching also has a guaranteed lower bound on the results. It achieves a (1/2)-approximation solution (i.e., the overall cohesion score will be at least 1/2 of the optimal cohesion score) as pointed out in [22].

Since the maximal weighted matching results in an overall good performance on time complexity and approximation rate, we used the maximal weighted matching in our empirical study for Algorithms 2 and 3. Readers may also choose other matching algorithms (such as the one described in [22]) to achieve slightly better approximation rates. However, the weighted matching is a replaceable module for our algorithms and it is not the focus of this work to build a fast and close-to-optimal weighted matching algorithm.

Compared to the time complexity of integrating ontologies by the dynamic programming approach as described in Algorithms 2 and 3, the heuristic approach described at the beginning of this section also has *O*(*n* ^2^) in the worst case. However, we conjecture that the heuristic approach has a much smaller average time complexity because, in each step, the heuristic approach may exclude a large number of matching opportunities.

### 2.2. Integrating Multiple Ontologies

In the previous section we proposed methods for integrating two ontologies. In some biomedical applications [23, 24], we are interested in the associations involving more than two objects. Integration of multiple ontologies of these objects will generate an innovative view on these complex relationships. Similar to the basic problem formulation, we can formulate the multiple ontology integration as follows.

Given *k* ontology trees *T*_1_, *T*_2_,…, *T*_*k*_ and a closeness matrix *M*_*ij*_ for any two trees *T*_*i*_ and *T*_*j*_, how can we efficiently generate an integrated ontology tree *T*_1,2,…,*k*_ meeting the following criteria?

(1) For any two vertices *x* and *y* in a tree *T*_*i*_, their lowest common ancestor LCA_*T*_*i*__(*x*, *y*) is contained by LCA_*T*_1,2,…,*k*__(*x*, *y*).
(2) It holds that argmax_*T*_1,2,…,*k*__ *f*(*T*_1,2,…,*k*_) = ∑_*X*∈*V*(*T*_*AB*_)_∑_*u*∈*X*,*v*∈*X*,*σ*(*u*)<*σ*(*v*)_ *M*_*σ*(*u*),*σ*(*v*)_(*u*, *v*). Here *M*_*σ*(*u*),*σ*(*v*)_(*u*, *v*) is the entry value in the closeness matrix for two vertices *u* and *v* (one from tree *T*_*σ*(*u*)_ and the other from tree *T*_*σ*(*v*)_) contained in the node *X*. For a vertex *v* from an original ontology, *σ*(*v*) is defined as its original ontology ID.

Again, we name the function *f*_*T*_1,2,…,*k*__ the cohesion of the integrated ontology *T*_1,2,…,*k*_. For each node *X* in the integrated ontology, we define its weight as *weight*(*X*) = ∑_*u*∈*X*,*v*∈*X*,*σ*(*u*)<*σ*(*v*)_ *M*_*σ*(*u*),*σ*(*v*)_(*u*, *v*). Correspondingly, we define function *g*(*T*_1_, *T*_2_,…, *T*_*k*_) = max_*T*_1,2,…,*k*__(∑_*X*∈*V*(*T*_1,2,…,*k*_)_ *weight*(*X*)) as the maximum cohesion function for integrating the ontologies *T*_1_, *T*_2_,…, *T*_*k*_. As we can see in the above formulation, the overall cohesion score of integration is the summed weight of each node, which is the sum of pairwise closeness scores.

The formulation of multiple ontology integration is similar to the basic version, and it is not difficult to show that optimal structures described in Lemmas 1 and 2 can be extended to a high dimension. However, the extension of algorithms described in Section 2.1.2 for integrating two ontologies is not feasible for solving the multiple ontology integration. This is because if we need to extend Algorithms 2 and 3 to this problem, we need to build a cohesion matrix of *k* dimensions. It implies that we need at least *O*(*n* ^*k*^) operations to fill up the score matrix assuming the size of an ontology is *O*(*n*). This is clearly not acceptable for high dimensional ontology integration.

#### 2.2.1. Greedy Approach

From the above discussion we can see that direct extension of Algorithms 2 and 3 for integrating two ontologies is practically not feasible for integrating a large number of ontologies. However, we can still use these algorithms for integrating multiple ontologies, by iteratively integrating two ontologies and generating a new closeness matrix. Given the ontologies *T*_1_, *T*_2_,…, *T*_*k*_, we can first integrate *T*_1_ and *T*_2_ into *T*_1,2_ and then build the closeness matrix between *T*_1,2_ and *T*_3_ using the relationship matrices between *T*_1_ and *T*_2_ and between *T*_1_ and *T*_3_. Specifically, assume *X* is a node on the integrated ontology *T*_1,2_, and *X* contains a vertex *a* from *T*_1_ and a vertex *b* from *T*_2_. Then, the entry (*X*, *c*) of the closeness matrix between *T*_1,2_ and *T*_3_ is *M*_*T*_1,2_,*T*_3__(*X*, *c*) = *M*_*T*_1_,*T*_3__(*a*, *c*) + *M*_*T*_2_,*T*_3__(*b*, *c*). After the new closeness matrix is generated, we can continue integrating *T*_1,2_ and *T*_3_ into *T*_1,2,3_ and generating another new closeness matrix. We will eventually get the integrated ontology *T*_1,2,…,*k*_ by repeating the above process. To facilitate the following discussions, we name the above approach the* basic multiple integration approach*.

**Figure 4:**
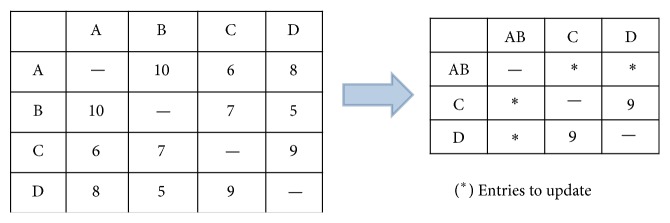
An example of the InterOntology matrix's change at the first iteration in Algorithm 4 for integrating four ontologies.

Although the basic multiple integration approach can finish integrating multiple ontologies, it blindly integrates ontologies without using any cohesion information between ontologies that may lead to a better integration result. To improve the basic multiple integration, we propose a greedy approach that uses the cohesion information between ontologies to guide the integration. The basic steps of the greedy approach are outlined in Algorithm 4. To facilitate the understanding of Algorithm 4, we use Figure 4 to illustrate an example of the InterOntology matrix's change at the first iteration of integrating four ontologies A, B, C, and D.

The key idea in Algorithm 4 is to maintain an InterOntology matrix which guides the integration. Initially, this matrix is filled with the overall cohesion score of every pair of ontologies. In each step, this matrix is updated with overall cohesion scores between newly integrated ontology and existing active ontologies. The integration will take place between two active ontologies which have the highest score in the InterOntology matrix.

When we used the overall cohesion score between two ontologies to update the InterOntology matrix, we observed an interesting phenomenon that the integration in most cases is a process continuously expanding an integrated ontology. Consequently, the greedy approach is likely to yield a result similar to the basic approach.

This phenomenon can be explained by the definition of maximum cohesion function, which takes into account all pairwise closeness between merged terms. Thus, the more ontologies contained in an integrated ontology are, the more likely it will have high overall cohesion scores with other ontologies. As a result, it creates unfairness for the integration selection. To fix this issue, we use the adjusted overall cohesion scores in updating the InterOntology matrix as follows.

Given an ontology *T*_*X*_ and an ontology *T*_*Y*_ where *X* and *Y* are nonempty sets of ontology IDs, we define the adjusted cohesion score between *T*_*X*_ and *T*_*Y*_ as *AdjCoh*(*T*_*X*_, *T*_*Y*_) = ∑_*Z*∈*V*(*T*_*XY*_)_∑_*x*∈*Z*,*y*∈*Z*,*σ*(*x*)∈*X*,*σ*(*y*)∈*Y*_ *M*_*σ*(*x*),*σ*(*y*)_(*x*, *y*)/|*X*||*Y*|, where *T*_*XY*_ is the integrated ontology built by Algorithms 2 and 3. The adjusted cohesion score is in fact the weight increase by integrating *T*_*X*_ and *T*_*Y*_, divided by the size of *X* times the size of *Y*; that is, *AdjCoh*(*T*_*X*_, *T*_*Y*_) = ∑_*Z*∈*V*(*T*_*XY*_)_(*weight*(*Z*)) − ∑_*X*∈*V*(*T*_*X*_)_(*weight*(*X*)) − ∑_*Y*∈*V*(*T*_*Y*_)_ *weight*(*Y*)/|*X*||*Y*|. For each node merging, closeness scores will be added to the total weight when the merging takes place between vertices from ontology set *X* and vertices from ontology set *Y*. Thus, the weight increase by integrating *T*_*X*_ and *T*_*Y*_ is proportional to the number of ontologies in *X* times the number of ontologies in *Y*, and consequently we averaged the weight increase by |*X*||*Y*|.

#### 2.2.2. Fast Approximation Algorithm

Although the basic multiple integration and the greedy multiple integration approaches discussed above are able to integrate multiple ontologies, none of them provide any guarantee on the results in comparison with the optimal solutions. By studying the maximum cohesion scores between ontologies under a graph setting, we identified an approximation structure and developed an approximation algorithm for integrating multiple ontologies. We name it fast approximation algorithm because it not only has a lower bound on the results, but also runs faster than the greedy multiple integration algorithm proposed above.

The fast approximation algorithm for integrating multiple ontologies is sketched in Algorithm 5. It only calculates the maximum cohesion score between every pair of ontologies once during the initial stage, and uses this information throughout the integration process even after it becomes stale. More importantly, this approach not only saves the time for recalculating the maximum cohesion scores, but also provides a lower bound guarantee as stated in Theorem 5, whose correctness is built on two important lemmas (Lemmas 6 and 7) which will be described subsequently.

**Theorem 5 .**

The tree weight of the integrated tree *T*_1,2,…,*k*_ obtained by FastMultiInt algorithm is at least 1/(*k* − 1) the weight of the optimal solution.

**Proof**

We will use Lemmas 6 and 7 to prove this theorem. The proofs of Lemmas 6 and 7 are provided after the proof of this theorem. To facilitate the proof of this theorem, we build a fully connected weighted graph *𝒢* in which each node corresponds to a tree for integration, and the weight of each edge corresponds to the weight increase (initially, this is the cohesion score) for integrating the corresponding trees. According to Lemma 6, *g*(*T*_1_, *T*_2_,…, *T*_*k*_) (i.e., optimal cohesion score) is no more than the summed weight of edges in *𝒢* (Claim  1).

**Figure 5:**
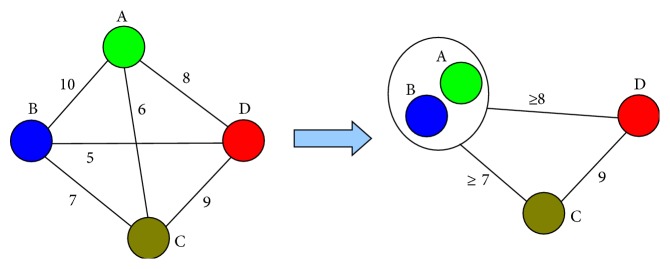
An illustration of vertex/edge contraction and weight updates in an iteration of Algorithm 5. Each vertex represents an ontology.

Given *𝒢*, the integration by Algorithm 5 is a process of *k* − 1 node contractions. After each contraction, the adjacent edge weights (cohesion scores) will be updated accordingly. According to Lemma 7, the weight of an updated edge will only increase over (or at least remain the same as) the maximum weight of the two contracted edges (Figure 5 provides an illustration of vertex/edge contraction and weight updates.) Thus, the overall cohesion score of the integration by Algorithm 5 is no less than the weight of the maximum spanning tree of *𝒢* (Claim  2).

It is easy to see that the weight of a maximum spanning tree is no less than 1/(*k* − 1) of the summed edge weight of *𝒢*, given the simple observation that each edge in *𝒢* is either an edge of the maximum spanning tree or adjacent to an edge of the maximum spanning tree with an equal or heavier weight (Claim  3).

Combining Claims 1, 2, and 3, we complete the proof of this theorem.

**Lemma 6 .**

It holds that *g*(*T*_1_, *T*_2_,…, *T*_*k*_) ≤ ∑_1≤*i*<*j*≤*k*_ *g*(*T*_*i*_, *T*_*j*_).

**Proof**

According to the problem definition,

(3) gT1,T2,…,Tk =max∑X∈VT1,2,…,kweightX =max⁡∑X∈VT1,2,…,k ∑u∈X,v∈X,σu<σvMσu,σvu,v =∑1≤i<j≤kfT1,2,…,kTi,j≤∑1≤i<j≤kgTi,Tj.

*f*_*T*_1,2,…,*k*__(*T*_*i*,*j*_) is the cohesion score of *T*_*i*,*j*_ whose integration is induced from *T*_1,2,…,*k*_.

**Lemma 7 .**

It holds that *g*(*T*_*P*,*Q*_, *T*_*S*_) − *f*(*T*_*P*,*Q*_) ≥ max(*g*(*T*_*P*_, *T*_*S*_), *g*(*T*_*Q*_, *T*_*S*_)).

**Proof**

According to the problem definition, integrating *T*_*P*,*Q*_ with *T*_*S*_ will result in an integrated tree *T*_*U*_ where *U* = *P* ∪ *Q* ∪ *S*, and *g*(*T*_*P*,*Q*_, *T*_*S*_) = max_*T*_*U*__(∑_*X*∈*V*(*T*_*U*_)_ *weight*(*X*)) = max_*T*_*U*__(*f*(*T*_*P*,*S*_) + *f*(*T*_*Q*,*S*_) + *f*(*T*_*P*,*Q*_)), where *T*_*P*,*S*_, *T*_*Q*,*S*_, and *T*_*P*,*Q*_ are induced from *T*_*U*_. Since *T*_*P*,*Q*_ has been determined, we have *g*(*T*_*P*,*Q*_, *T*_*S*_) = max_*T*_*U*__(*f*(*T*_*P*,*S*_) + *f*(*T*_*Q*,*S*_)) + *f*(*T*_*P*,*Q*_). Without loss of generally, let us assume max(*f*(*T*_*P*,*S*_)) ≥ max(*f*(*T*_*Q*,*U*_)). Then, by restricting the integration between *T*_*P*_ and *T*_*S*_ in *T*_*U*_ following the integration that leads to argmax_*T*_*P*,*S*__ *f*(*T*_*P*,*S*_), we will get a cohesion score no less than *g*(*T*_*P*_, *T*_*S*_). Thus, we complete the proof for *g*(*T*_*P*,*Q*_, *T*_*S*_) − *f*(*T*_*P*,*Q*_) ≥ max(*g*(*T*_*P*_, *T*_*S*_), *g*(*T*_*Q*_, *T*_*S*_)).

#### 2.2.3. Time Complexity Analysis

For the fast approximation algorithm (Algorithm 5), the time complexity for generating graph *𝒢* (calculating the overall cohesion score for every pair of ontologies) is *O*(*k* ^2^ *n* ^2^log*n*), assuming we use maximal weighted matching. Each integration will take *O*(*n* ^2^log*n*) with an update of at most *k* closeness matrices which takes *O*(*k∗n* ^2^). There are at most *k* integrations; therefore the total time complexity is still *O*(*k* ^2^ *n* ^2^log*n*).

Following the above analysis, we conclude that the time complexity of the greedy multiple integration algorithm is the same as the fast approximation algorithm. However, it requires updating of InterOntology matrix which takes an excessive *O*(*k* ^2^ *n* ^2^log*n*) time. The empirical study shows that the fast approximation algorithm is much faster than the greedy multiple integration algorithm.

Finally, it is easy to see that the basic multiple integration approach takes *O*(*kn* ^2^log*n*) time and is the fastest, but its overall cohesion scores are the worst as we will see in the empirical study.

#### 2.2.4. Limitations

Integrating multiple ontologies may face two potential problems in real applications. First, how can we efficiently generate a closeness matrix for every pair of ontologies to be integrated? Our current method kDLS or onGrid is efficient for generating the closeness matrix for one pair of ontologies in most cases, but not efficient enough for generating closeness matrices for many pairs of ontologies. Second, not every pair of ontologies can be meaningfully integrated. It remains a problem to efficiently identify the feasibility of integrating a pair of ontologies. Therefore, the main purpose of Section 2.2 is to demonstrate that our proposed approach can be extended to integrate multiple ontologies, and we use synesthetic datasets in Section 3.3 to study the performance of algorithms proposed in Section 2.2.

## 3. Results and Discussion

We would like to study the performances of the proposed ontology integration methods by experiments on both real and synthetic datasets. We implemented five approaches in C++:

(1) Heuristic: heuristic approach for integrating two ontologies as described in Section 2.1.1;
(2) Approximate: approximate approach (Algorithms 2 and 3) with maximal weighted matching for guaranteeing the (1/2)-approximation rate;
(3) Basic: basic multiple integration approach as described in Section 2.2.1;
(4) Greedy: greedy multiple integration approach (Algorithm 4);
(5) FastApproximate: fast approximation multiple integration approach (Algorithm 5).

In the following, we report our study on the performances of (1) and (2) for integrating two ontologies on real datasets and (3), (4), and (5) for integrating multiple ontologies on synthetic datasets. All the experiments are carried out on a Linux cluster with 2.4 GHz AMD Opteron processors.

### 3.1. Integrating a Pair of Ontologies

The knowledge of drug-gene relationships is desirable in many pharmacology applications [25, 26]. By integrating the gene ontology and the drug ontology, we will be able to obtain rich information on the associations between drugs and genes under the ontology structures. Thus, in this set of experiments, we simulate real world knowledge discovery applications by integrating two real ontologies, gene ontology (GO) and National Drug File Reference Terminology (NDFRT). Both were obtained from the Unified Medical Language System (version: 2012AA). The closeness matrices between GO terms and drug terms were generated using onGrid [27] with a 4-neighborhood broadcast range (i.e., *k* = 4 with regard to [7]). onGrid follows the kDLS approach [7] and measures the closeness between two concepts based on the discovered paths (with length greater than one) between them. However, unlike kDLS, onGrid takes into consideration of concept semantic types in the closeness measurement. In the study performed in [27], the advantages of onGrid over kDLS are well illustrated.

The overall cohesion scores of Heuristic and Approximate on integrating GO and NDFRT are listed in Table 1. To observe the integration over the ontology size change, in each experiment we use the ontology tree structure from the root to the specified depth (first column in Table 1) for integration. The sizes of the ontology terms involved in the integration are listed in the second and third columns of Table 1.

Recall in Section 2.1.1; *β* ^rdepth(*b*)^ is used to regulate the selection of vertices from high depths. Thus, we tested the Heuristic under *β* = 1 (depth information is nullified) and *β* = 100 (the vertex depth plays a critical role in the selection). Since nonleaf vertices in these datasets have around 6 children on average, we heuristically add a set of experiments by setting *β* = 6 so that *β* ^rdepth(*b*)^ will be close to the number of vertices excluded from the future integration.

From Table 1, we can see that Heuristic performs better when using the depth information to regulate the selection of vertices. However, Approximate is much better than Heuristic at all settings. Compared to the best cohesion scores of Heuristic in each row of Table 1, Approximate constructs an integrated ontology with the overall cohesion score ranging from 5.4 times to 109.9 times that of Heuristic. This clearly demonstrates the effectiveness of the proposed Approximate approach. Nevertheless, the heuristic approach has a much faster average running time as a result of excluding a large number of matching opportunities in each step.

Although the running time of Approximate is longer than Heuristic, it takes less than two hours to finish integrating two ontologies with about 16*k* and 33*k* vertices. Most of the biomedical ontologies are smaller than or similar to these sizes and Approximate approach will benefit the association study of these ontologies. For extremely large ontology pairs in which Approximate is unable to finish the integration within a reasonable time, Heuristic may provide a quick view on their integration.

### 3.2. Understanding the Merged Ontology Terms

To understand what terms are merged in integrating real ontologies, we use the integration of GO and NDFRT at depth 6 as an example. Tables 2 and 3 list the top 5 pairs of merged terms (sorted by their closeness scores) by Heuristic and Approximate, respectively. As mentioned above, these scores are from the closeness matrix generated by onGrid based on the discovered paths between them. For example, “C1155065:T-Cell Activation −  − is_physiologic_effect_of_chemical_or_drug −  −  > C0393002:Carcinoembryonic Antigen Peptide 1 −  − has_target −  −  > C0007082:Carcinoembryonic Antigen” is such a path.

From Tables 2 and 3 we can observe that the Approximate algorithm merges terms with much higher similar scores than the Heuristic algorithm. Quite interestingly, we observed that the top ranked merging in Table 3 is between “biological_process” and “VITAMINS.” The “biological_process” is an abstract term which is very close to the root of the GO ontology. Such a fact suggests that the top level terms will likely preempt the merging choices over their descendants. As a result of this greedy approach, the Heuristic algorithm will end at a local optimum which is far from being optimal.

A snapshot of ontology integration by Approximate as shown in Figure 10 provides a good insight on the algorithm work. In each bracket of two merged terms, the left part is the closeness score and the right part is the cohesion score. We can observe that most closeness scores are zero or close to zero, while the corresponding cohesion scores are much higher. This is understandable because the snapshot is primarily on the top level terms of both ontologies. For these terms, they have a large number of subclass (descendant) terms, and optimizing the integration of their subclass terms far outweighs integrating of themselves. The result of such integration provides novel knowledge of association between ontology terms. That is, even if two terms are not that close according to some closeness measurement, they can be structurally associated under their ontology context. For example, the GO term “biological_process” is merged with the NDFRT term “chemical ingredients”; even their closeness score is zero from the onGrid output. However, such integration is interesting because it shows that the merging is trying to link the chemical compounds with the biological/cellular processes so that corresponding associations between the cellular processes and chemical structures can be established. This demonstrates the purpose of integrating two ontologies, that is, identifying associations with respect to both term similarities and structural contexts.

In fact, there are multiple studies to justify the structural associations seen in Figure 10, such as the association between “signaling” and “carbohydrates” [28] and the association between “extracellular region part” and “skin and connective tissue diseases” [29].

In addition, we have noticed a number of meaningful integrations between GO terms and neurological terms in the NDFRT. For example, synapse is a brain related structure and the term “symmetric synapse” is associated with “trauma,” and the term “asymmetric synapse” is associated with “brain neoplasms.” Similarly, it is reasonable to see that “neuronal RNA granule” is integrated with “granulomatous disease,” a granule associated disease. As another example, it is very interesting to notice that “zyxin” is associated with “cell adhesion involved in heart morphogenesis” and that provides a link with the formation of heart.

The above observations suggest a novel way of using our ontology integration method to perform association studies between biomedical concepts.

### 3.3. Integrating Multiple Ontologies

In the following experiments we will study the performances of Basic, Greedy, and FastApproximate in integrating multiple ontologies. All the three approaches are built upon Approximate, which performs very well in the previous study for integrating two real ontology datasets.

We use two sets of synthetic datasets in this study. In the first set of datasets, we fix the number of ontologies to be 10 and vary the size of each ontology from 100 to 1000. In the second set of datasets, we fix the size of each ontology to be 100 and vary the number of ontologies from 10 to 100. All the ontologies are randomly generated by constructing a minimal spanning tree from a random matrix. The relationship matrix between every pair of ontologies is also randomly generated with entry values ranging from 0 to 1. For each experiment, we generate 10 random datasets and the results reported in the following are the average results over the 10 random datasets.

**Figure 6:**
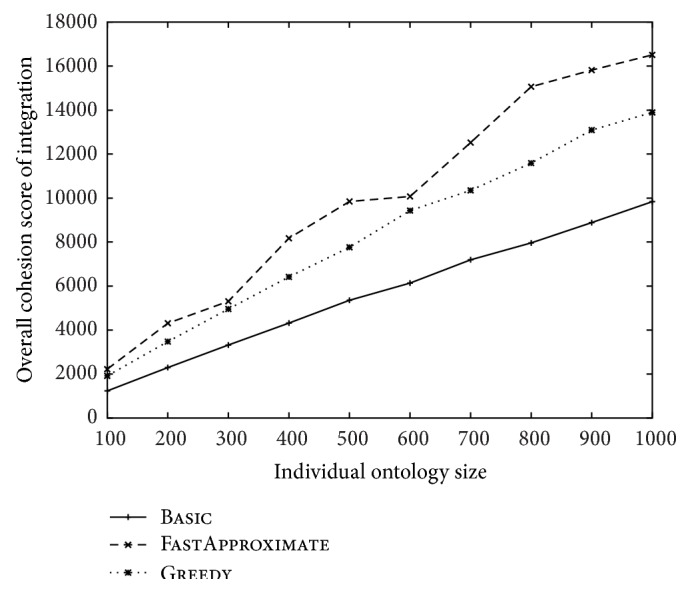
The change of overall cohesion score over the increase of the size of each ontology. The number of ontologies is fixed at 10.

**Figure 7:**
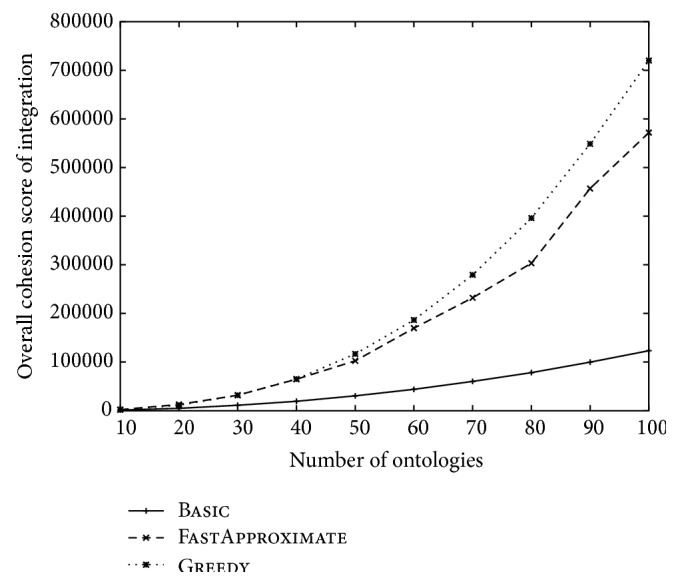
The change of overall cohesion score over the increase of the number of ontologies. The size of each ontology is fixed at 100.

The overall cohesion scores of the three approaches over different ontology sizes and over different numbers of ontologies were reported in Figures 6 and 7, respectively. FastApproximate outperforms all the other approaches in Figure 6, which is consistent with the analysis of its approximation rate. However, Greedy slightly outperforms FastApproximate in Figure 7 especially when the ontology number is large. This is understandable because when the number of ontology (*k*) increases, the approximation rate (as stated in Theorem 5) decreases and becomes less significant. This result also justifies the choice of adjusted cohesion score for Greedy as described at the end of Section 2.2.1.

**Figure 8:**
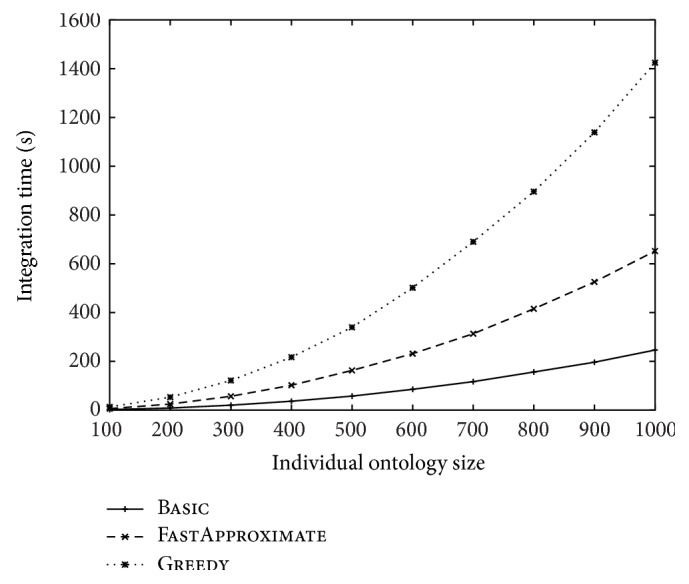
The change of integration time over the increase of the size of each ontology. The number of ontologies is fixed at 10.

**Figure 9:**
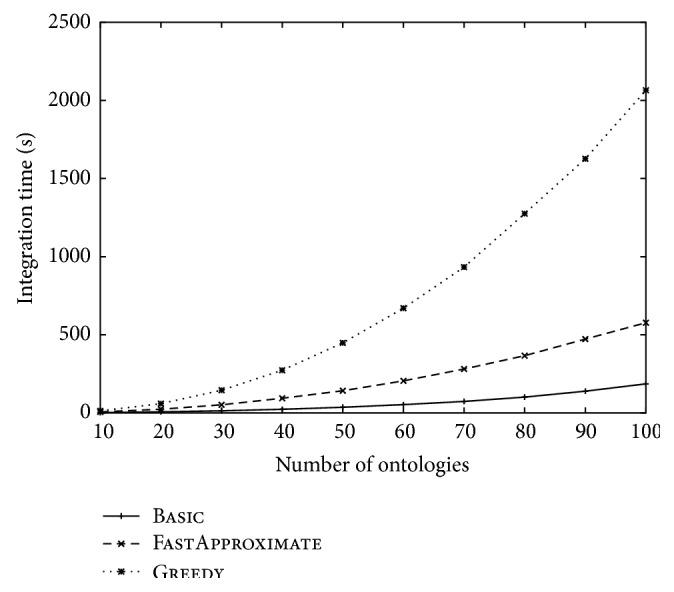
The change of integration time over the increase of the number of ontologies. The size of each ontology is fixed at 100.

**Figure 10:**
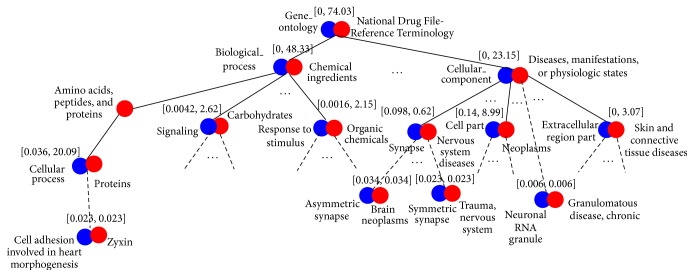
A snapshot of ontology integration between GO and NDFRT by Approximate (depth = 6). Blue nodes are terms from GO and red nodes are terms from NDFRT. Solid lines are edges and dashed lines are paths consisting of 1 or more edges. In each bracket, the left part is the closeness score and the right part is the cohesion score.

The integration time of the three approaches over different ontology sizes and over different numbers of ontologies was reported in Figures 8 and 9, respectively. These figures are consistent with the time complexity analysis given in Section 2.2.3. In particular, we noticed that the integration time of Greedy deteriorates sharply over the ontology number increase. In contrast, FastApproximate is much more scalable and has a time curve similar to Basic.

These results suggest that FastApproximate has the best overall performance in integrating multiple ontologies.

## 4. Conclusions

In this work, we started with a basic problem on integrating a pair of ontology tree structures with a given closeness matrix, and later we advanced the basic problem to the problem of integrating large number of ontologies. We proved optimal structures in the basic problem and developed both optimal and efficient approximation solutions. Although the multiple ontology integration problem has similar optimal structures, it is not feasible to extend the optimal and efficient approximation solutions for the basic problem to efficiently handle multiple ontology integration. To tackle the challenge of integrating a large number of ontologies, we developed both an effective greedy approach and a fast approximation approach. The empirical study not only confirms our analysis on the efficiency of the proposed method, but also demonstrates that our method can be used effectively for biomedical association studies.

## Figures and Tables

**Algorithm 1:**
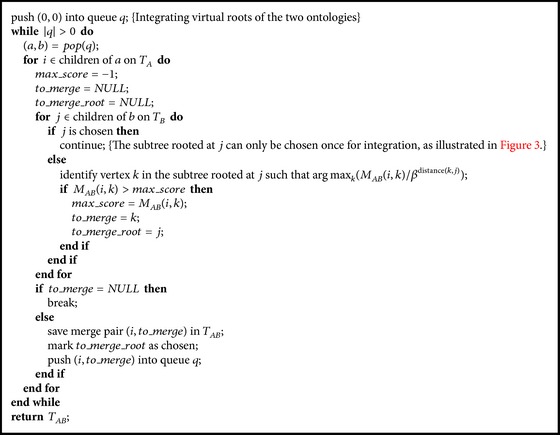
He uristicMerge(*T*_*A*_, *T*_*B*_, *M*_*AB*_).

**Algorithm 2:**
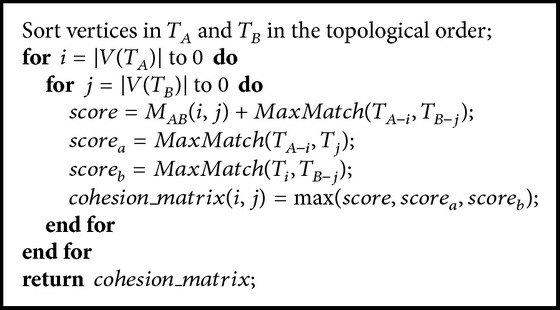
Bu ildCohesionMatrix(*T*_*A*_, *T*_*B*_).

**Algorithm 3:**
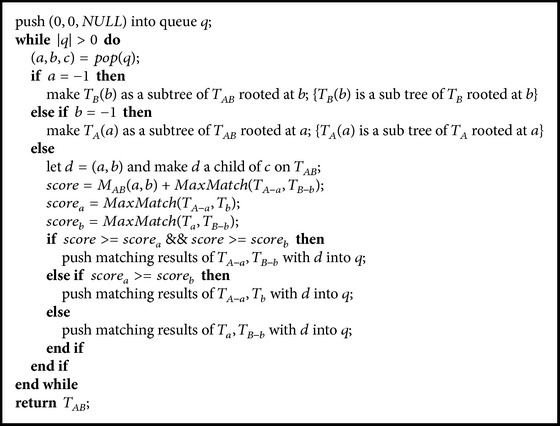
Bu ildNewOnto(cohesion_matrix, *T*_*A*_, *T*_*B*_).

**Algorithm 4:**
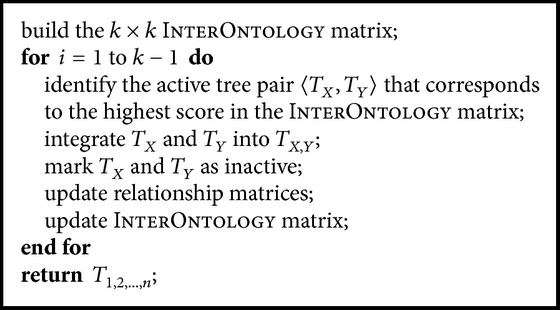
Gr eedyMultiInt(*𝒯* = {*T*_1_, *T*_2_,…, *T*_*k*_}).

**Algorithm 5:**
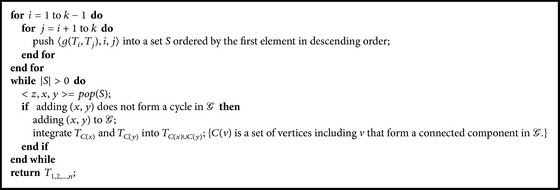
Fa stMultiInt(*𝒯* = {*T*_1_, *T*_2_,…, *T*_*k*_}).

**Table 1:** Cohesion scores of integrating real datasets.

Depth	GO term number	NDFRT term number	Cohesion scores			
			He uristic β = 1	Heuristic β = 6	Heuristic β = 100	Approximate
3	66	6004	0.0505331	0.229958	0.21999	1.24696
4	710	6972	0.290392	0.284363	1.46585	9.3835
5	5355	14582	0.285923	1.37714	0.528289	33.9056
6	16231	32841	0.307941	0.341588	0.673406	74.0293

**Table 2:** Top 5 matched terms by the Ap proximate algorithm (depth = 6).

Rank	GO terms	NDFRT terms	Closeness score
1	C1135918 smooth muscle contractile fiber	C0282606 muscle neoplasms	1.63205
2	C0010813 cytokinesis	C0086376 GTP-binding proteins	1.15967
3	C0027747 axon terminus	C0030584 parovarian cyst	1.13352
4	C1155065 T cell activation	C0007082 carcinoembryonic antigen	1.00879
5	C0007595 cell growth	C0294028 BRCA2 protein	0.945284

**Table 3:** Top 5 matched terms by the He uristic algorithm (depth = 6, β = 100).

Rank	GO terms	NDFRT terms	Closeness score
1	C0031845 biological_process	C0042890 VITAMINS	0.244862
2	C1166607 cellular_component	C1657248 apoptosome	0.142857
3	C0027540 tissue death	C0065932 MENADIOL	0.0434219
4	C0025519 metabolic process	C0042849 VITAMIN B	0.0370248
5	C0030012 oxidation-reduction process	C0027996 NICOTINIC ACID	0.0327109

## References

[1] McGuinness D. L., van Harmelen F., Smith M. K.

[2] Bodenreider O. (2004). The unified medical language system (UMLS): integrating biomedical terminology. *Nucleic Acids Research*.

[3] Wu X., Jiang R., Zhang M. Q., Li S. (2008). Network-based global inference of human disease genes. *Molecular Systems Biology*.

[4] Linghu B., Snitkin E. S., Hu Z., Xia Y., DeLisi C. (2009). Genome-wide prioritization of disease genes and identification of disease-disease associations from an integrated human functional linkage network. *Genome Biology*.

[5] Botstein D., Risch N. (2003). Discovering genotypes underlying human phenotypes: past successes for mendelian disease, future approaches for complex disease. *Nature Genetics*.

[6] Pinto H. S., Martins J. P. A methodology for ontology integration.

[7] Xiang Y., Lu K., James S. L., Borlawsky T. B., Huang K., Payne P. R. O. (2012). K-Neighborhood decentralization: a comprehensive solution to index the UMLS for large scale knowledge discovery. *Journal of Biomedical Informatics*.

[8] Dutkowski J., Kramer M., Surma M. A. (2013). A gene ontology inferred from molecular networks. *Nature Biotechnology*.

[9] Navigli R., Velardi P., Gangemi A. (2003). Ontology learning and its application to automated terminology translation. *IEEE Intelligent Systems*.

[10] Jannink J., Wiederhold G. Thesaurus entry extraction from an on-line dictionary.

[11] Papatheodorou C., Vassiliou A., Simon B. Discovery of ontologies for learning resources using word-based clustering.

[12] Rubin D. L., Hewett M., Oliver D. E., Klein T. E., Altman R. B. (2001). Automating data acquisition into ontologies from pharmacogenetics relational data sources using declarative object definitions and xml. *Pacific Symposium on Biocomputing*.

[13] Noy N. F. (2004). Semantic integration: a survey of ontology-based approaches. *ACM SIGMOD Record*.

[14] Abels S., Haak L., Hahn A. Identification of common methods used for ontology integration tasks.

[15] Gangemi A., Pisanelli D., Steve G. (1998). Ontology integration: experiences with medical terminologies. *Formal Ontology in Information Systems*.

[16] Noy N. F., Musen M. A. SMART: automated support for ontology merging and alignment.

[17] Doan A., Madhavan J., Domingos P., Halevy A. Learning to map between ontologies on the semantic web.

[18] Xie J., Liu F., Guan S.-U. (2011). Tree-structure based ontology integration. *Journal of Information Science*.

[19] Calvanese D., De Giacomo G., Lenzerini M. A framework for ontology integration.

[20] Udrea O., Getoor L., Miller R. J. Leveraging data and structure in ontology integration.

[21] Kuhn H. W. (1955). The Hungarian method for the assignment problem. *Naval Research Logistics Quarterly*.

[22] Drake Vinkemeier D. E., Hougardy S. (2005). A linear-time approximation algorithm for weighted matchings in graphs. *ACM Transactions on Algorithms*.

[23] Wood A. J. J., Evans W. E., McLeod H. L. (2003). Pharmacogenomics—drug disposition, drug targets, and side effects. *The New England Journal of Medicine*.

[24] Altman R. B. (2007). Pharmgkb: a logical home for knowledge relating genotype to drug response phenotype. *Nature Genetics*.

[25] Kim R. B., O'Shea D., Wilkinson G. R. (1995). Interindividual variability of chlorzoxazone 6-hydroxylation in men and women and its relationship to CYP2E1 genetic polymorphisms. *Clinical Pharmacology & Therapeutics*.

[26] Ferraro T. N., Buono R. J. (2005). The relationship between the pharmacology of antiepileptic drugs and human gene variation: an overview. *Epilepsy and Behavior*.

[27] Albin A., Ji X., Borlawsky T. B. (2014). Enabling online studies of conceptual relationships between medical terms: developing an efficient web platform. *JMIR Medical Informatics*.

[28] Chandrasekaran S., Dean J. W., Giniger M. S., Tanzer M. L. (1991). Laminin carbohydrates are implicated in cell signaling. *Journal of Cellular Biochemistry*.

[29] Uitto J., Kouba D. (2000). Cytokine modulation of extracellular matrix gene expression: relevance to fibrotic skin diseases. *Journal of Dermatological Science*.

